# Real time PCR detection of common *CYP2D6* genetic variants and its application in a Karen population study

**DOI:** 10.1186/s12936-018-2579-8

**Published:** 2018-11-15

**Authors:** Kanokpich Puaprasert, Cindy Chu, Naowarat Saralamba, Nicholas P. J. Day, Francois Nosten, Nicholas J. White, Arjen M. Dondorp, Mallika Imwong

**Affiliations:** 1 Department of Molecular Tropical Medicine and Genetics, Faculty of Tropical Medicine, Mahidol University, Bangkok, Thailand; 2 Shoklo Malaria Research Unit, Mahidol-Oxford Tropical Medicine Research Unit, Bangkok, Thailand; 3 Mahidol Oxford Tropical Medicine Research Unit, Faculty of Tropical Medicine, Mahidol University, Bangkok, Thailand; 4 Centre for Tropical Medicine and Global Health, Nuffield Department of Medicine, University of Oxford, Oxford, UK

**Keywords:** Malaria, Primaquine, *CYP2D6*, Karen

## Abstract

**Background:**

*Plasmodium vivax* malaria is characterized by relapses arising from the hypnozoite stages in the liver. The only currently registered drug for radical treatment to prevent relapse is primaquine. Primaquine, a prodrug, requires metabolism through the liver cytochrome CYP2D6 isoenzyme to its active metabolite. Mutations in the *CYP2D6* gene may thus affect primaquine efficacy. A SNPs genotyping technique was developed to characterize the *CYP2D6* genetic variants and tested this in the patients with *Plasmodium vivax* infection collected in a Karen population on the Thailand–Myanmar border, where *P. vivax* malaria is endemic.

**Methods:**

Direct sequencing of PCR-reamplified products (DSP) was used to uncover exonic *CYP2D6* sequence variations. Subsequently, an allele-specific oligonucleotide probe real-time SNPs genotyping (ASO) assay was developed for rapid detection of the four clinically relevant *CYP2D6* variants occurring in this population. These two in-house developed assays were used to genotype *CYP2D6* mutations in blood samples obtained from 70 Karen adults.

**Results:**

Results showed a high degree of concordance between the DSP and ASO methods. Six *CYP2D6* point mutations were identified within the Karen population: C100T, C1039T, G1661C, G1846A, C2850T and G4180C, at frequencies of 0.43, 0.43, 0.76, 0.02, 0.32 and 0.76, respectively. The CYP2D6*2, *4, *5, *10 and *36 allelic frequencies were 0.33, 0.02, 0.03, 0.40 and 0.01, respectively. Alleles conferring an intermediate *CYP2D6* metabolizer phenotype comprised 46% of the total number of alleles.

**Conclusion:**

The newly developed ASO assay is a reliable and rapid tool for large-scale *CYP2D6* genotyping. The high frequency of the CYP2D6*10 allele in the Karen population warrants further assessment of its association with the radical curative efficacy of primaquine.

**Electronic supplementary material:**

The online version of this article (10.1186/s12936-018-2579-8) contains supplementary material, which is available to authorized users.

## Background

Primaquine (PQ), an 8-aminoquinoline, is currently the only widely available drug for radical treatment of *Plasmodium vivax* malaria. It is active against the dormant hypnozoite stages of the parasite in the liver responsible for relapse infections [1]. Primaquine itself is biologically inactive and requires biotransformation to active metabolites for its anti-hypnozoite effect. Two metabolic pathways have been identified, involving monoamine oxidase-A (MAO-A) and the cytochrome P450 CYP2D6 isoenzyme. Carboxyprimaquine which is generated via the MAO-A mediated pathway is the most abundant metabolite in plasma, but it is not considered hypnozoitocidal [2–4]. The active phenolic metabolites resulting from metabolism through CYP2D6 are likely to exert anti-malarial properties mediated by the production of oxidative stress through redox cycling [4–7]. A number of studies have shown that CYP2D6 plays a crucial role in the metabolic activation of primaquine [4, 8–11] and that mutations in *CYP2D6* can potentially affect primaquine efficacy [12, 13]. Accurate genotyping of the *CYP2D6* gene is difficult because of the presence of highly homologous pseudogenes and the highly polymorphic character of the gene. The *CYP2D8* and CYP*2D7* flanking pseudogenes display over 90% nucleotide sequence homology compared to the active *CYP2D6* gene [14, 15], potentially resulting in co-amplification during the gene amplification process [16, 17]. Consequently, multiple homologous PCR templates and mis-primed sequences containing several inactive mutations can easily result in incorrect genotype assignments [14, 18, 19]. In addition, there is wide genetic variability in *CYP2D6*, including single nucleotide substitutions, insertion/deletion, partial gene conversions [20], *CYP2D7/2D6* hybrid tandems [21], copy number variations (CNVs) [16, 22, 23] and complex structural rearrangements [17, 24, 25]. This has resulted in the characterization of over one-hundred *CYP2D6* variant alleles, which further complicates genotyping. Furthermore, the prevalence of allelic variants associated with impaired *CYP2D6* catalytic activity have been found to vary widely across ethnic populations [26–28]. In the present study, a *CYP2D6* genotyping protocol was developed to screen for variants with known clinical significance in Southeast Asia and tested the method on *P. vivax* infection samples collected from a Karen population on the Thailand–Myanmar border.

## Methods

### Study population and DNA preparation

Seventy anonymized blood samples were collected in the Karen population living in Tak, one of the western provinces in Thailand. All patients were diagnosed by microscopy of thick and thin blood smears, examined by qualified laboratory technicians. Only patients with *P. vivax* mono-infections were included in the study. Among vivax patients, 39 (56%) were females, the median age was 16 years old (IQR 11–24 years old), the median weight was 40 kg (IQR 18–50 kg), and the median parasitaemia was 4024 parasites/µl (IQR 1592–10,793 parasites/µl). Genomic DNA extraction was by the QIAamp DNA Mini Kit (Qiagen, Germany), according to the manufacturer’s guidelines. The isolated genomic DNA samples were stored at 4 °C until further processing. Allele designation followed the Human Cytochrome P450 (*CYP*) Allele Nomenclature Database (http://www.imm.ki.se/CYPalleles/) (Table 1). The study was approved by the Ethics Committee of the Faculty of Tropical Medicine, Mahidol University (EC Submission No.: TMEC 15-095).

**Table 1:** *CYP2D6* alleles in the Karen study population and related nucleotide and amino acid substitutions

Alleles	Nucleotide substitutions	Amino acid substitutions	Ref SNP ID
CYP2D6*1	Reference allele	Reference protein	–
CYP2D6*2	**C2850T**, G4180C	**R296C**, S486T	rs16947
CYP2D6*4	C100T, **G1846A**, G4180C	P34S, **splicing defect**, S486T	rs3892097
CYP2D6*5	Deletion of *CYP2D6* gene	Not applicable	Not applicable
CYP2D6*10	**C100T**, G4180C	**P34S**, S486T	rs1065852
CYP2D6*36	C100T, G4180C, **gene conversion to** ***CYP2D7*** **in exon 9**	P34S, S486T	–

The nucleotide and amino acid substitutions in bold letters denote key variations used to assign particular variant alleles, based on Gene bank accession number M33388.1. Unique allele names were assigned as described in the Human Cytochrome P450 (*CYP*) Allele Nomenclature Database (http://www.imm.ki.se/CYPalleles/)

### Primers and probes

**Fig. 1:**
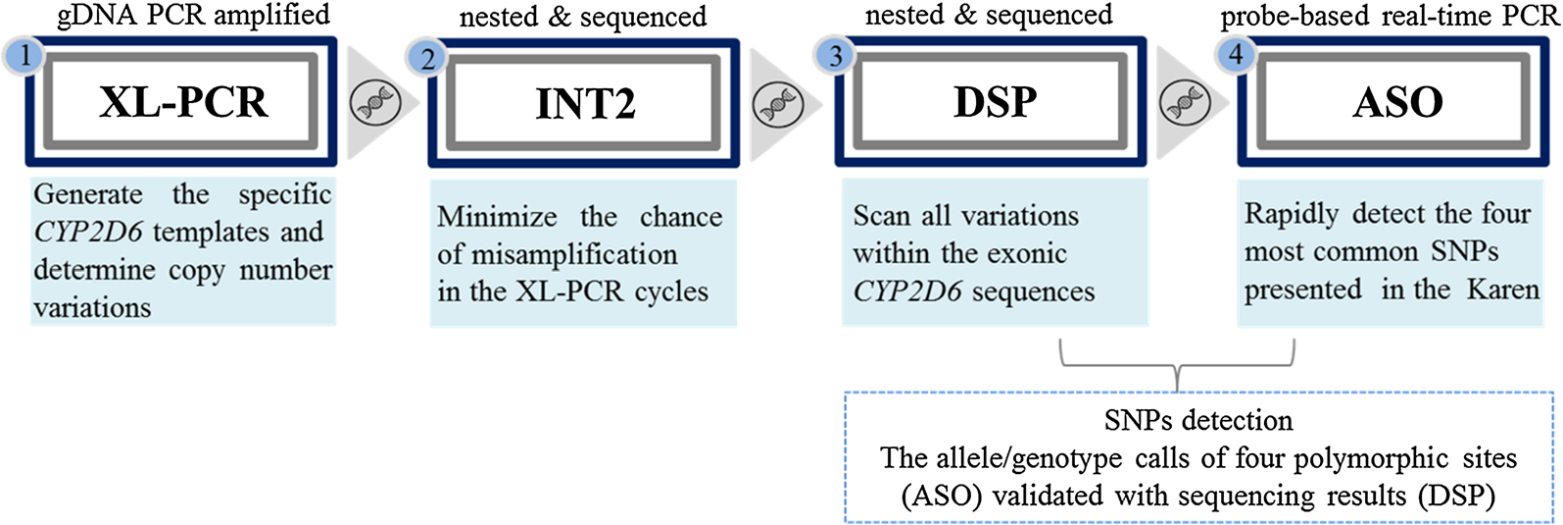
Summary of the assay development for assessment of common *CYP2D6* genetic variants relevant for the Karen study population

Two genomic sequences retrieved from NCBI, *CYP2D6* (GeneBank accession number M33388.1) and *CYP2D7/8* (GeneBank accession number M33387.1), were used as genomic reference templates. Optimal primer sequences of PCR-reamplified products were selected by Primer3 software version 0.4.0 and were then synthesized by Macrogen Inc. (Korea). Variant-specific primer and probe sets of real-time PCR were designed and supplied directly by Applied Biosystems (Thermal Fisher Scientific, Inc.). Wild-type and mutant *CYP2D6* probes were labeled at the 5′ end with VIC and FAM respectively, and both probes included a non-fluorescent quencher. The sequences of primers and probes of all PCRs performed in each step are listed in Table 2. Positive controls are well-characterized samples sequenced across the entire *CYP2D6* gene and redetected by real-time PCR assay. A summary of the genotyping method is provided in Fig. 1 and can be summarized in the following four steps.

**Table 2:** Primer and probe sequences used for detection of *CYP2D6* gene mutations

Analysis	Primer and probe names	Sequences (5′–3′)	Length (bp)	GC (%)	T_m_ (°C)	Amplicon size (bp)
XL-PCR [29]	DPKup	5′-GTTATCCCAGAAGGCTTTGCAGGCTTCA-3′	28	50.0	67.8	5100
	DPKlow	5′-GCCGACTGAGCCCTGGGAGGTAGGTA-3′	26	65.4	71.1	
	2D6dupl-F	5′-CCTGGGAAGGCCCCATGGAAG-3′	21	66.7	65.5	3500
	2D6dupl-R	5′-CAGTTACGGCAGTGGTCAGCT-3′	21	57.1	63.2	
	5′2D6*5	5′-CACCAGGCACCTGTACTCCTC-3′	21	61.9	62.7	3500
	3′2D6*5	5′-CAGGCATGAGCTAAGGCACCCAGAC-3′	25	60.0	67.9	
Int2	5′2D6Int2	5′-TTTTGCACTGTGGGTCCTC-3′	19	52.6	58.5	1101
	3′2D6Int2	5′-CAAGGTGGACACGGAGAAG-3′	19	57.9	58.4	
Direct sequencing of PCR-reamplified products, DSP	5′2D6Ex1	5′-GCACAGTCAACACAGCAGGT-3′	20	55.0	61.7	503
	3′2D6Ex1	5′-AATGCCCTTCTCCAGGAAGT-3′	20	50.0	59.2	
	5′2D6Ex2	5′-TTCCTCCATCACAGAAGGTG-3′	20	50.0	57.4	501
	3′2D6Ex2	5′-CTCCCTAGTGCAGGTGGTTT-3′	20	55.0	59.9	
	5′2D6Ex34	5′-GTCTTCCCTGAGTGCAAAGG-3′	20	55.0	59.1	754
	3′2D6Ex34	5′-AGTGGGGTCTCCTGGAATG-3′	19	57.9	58.9	
	5′2D6Ex56	5′-GAGGGACTTGGTGAGGTCAG-3′	20	60.0	60.0	794
	3′2D6Ex56	5′-GACACTCCTTCTTGCCTCCT-3′	20	55.0	59.6	
	5′2D6Ex7	5′-ATGAACTTTGCTGGGACACC-3′	20	50.0	59.0	505
	3′2D6Ex7	5′-CCAGCCCTGCCTATACTCTG-3′	20	60.0	59.9	
	5′2D6Ex89	5′-TCTAGTGGGGAGACAAACCAG-3′	21	52.4	59.3	802
	3′2D6Ex89	5′-CTGAGGAGGATGATCCCAAC-3′	20	55.0	57.7	
Allele-specific oligonucleotide probes real time SNPs genotyping, ASO						
C100T	5′2D6C100T	5′-CCTGGTGGACCTGATGCA-3′	18	61.1	59.5	73
	3′2D6C100T	5′-CCCGGGCAGTGGCA-3′	14	78.6	58.7	
	2D6C100T_WT	5′-CCTGGTG**G**GTAGCGTG-3′	16	69.0	51.1	
	2D6C100T_MT	5′-CCTGGTG**A**GTAGCGTG-3′	16	63.0	48.5	
G1846A	5′2D6G1846A	5′-GACCCCTTACCCGCATCTC-3′	19	63.2	60.1	73
	3′2D6G1846A	5′-GCTCACGGCTTTGTCCAAGA-3′	20	55.0	61.5	
	2D6G1846A_WT	5′-CCCCCA**G**GACGCC-3′	13	85.0	48.0	
	2D6G1846A_MT	5′-CCCCCA**A**GACGCC-3′	13	77.0	46.0	
C2850T	5′2D6C2850T	5′-CCTGAGAGCAGCTTCAATGATGA-3′	23	47.8	61.3	67
	3′2D6 C2850T	5′-CCATCCCGGCAGAGAACAG-3′	19	63.2	60.7	
	2D6C2850T _WT	5′-ACTATGC**G**CAGGTTC -3′	15	53.0	41.9	
	2D6C2850T _MT	5′-CACTATGC**A**CAGGTTC-3′	16	50.0	43.4	
G4180C	5′2D6G4180C	5′-CCACCATGGTGTCTTTGCTTTC-3′	22	50.0	60.9	67
	3′2D6G4180C	5′-GCACAGCACAAAGCTCATAGG-3′	21	52.4	60.4	
	2D6G4180C _WT	5′-CTGGTGA**G**CCCATCC-3′	15	67.0	47.4	
	2D6G4180C _MT	5′-CTGGTGA**C**CCCATCC-3′	15	67.0	47.4

### Determination of *CYP2D6* gene duplications/multiplications and gene deletions by extra-long range polymerase chain reaction (XL-PCR)

In a single tetra-primer XL-PCR mixture, two separate reactions including the *CYP2D6* gene duplications/multiplications and deletions, both producing a 3.5 kb long PCR fragment, were determined simultaneously with the amplification of a 5.1 kb fragment encompassing the entire *CYP2D6* gene. The generated *CYP2D6* fragments were then used as templates for genotyping as described previously [29].

### Discrimination of functional *CYP2D6* and nonfunctional *CYP2D8* and *CYP2D7* genes by intron 2 sequencing (INT2)

In each XL-PCR run, the newly produced fragments were randomly sampled to be reamplified and sequenced in the intron 2 region. All nested INT2 reactions were carried out on a Mastercycler pro (Eppendorf, Hamburg, Germany). The XL-PCR products were reamplified in 25 µl reaction volumes containing 19.55 µl of nuclease-free water, 2.5 µl of 1× Master Mix buffer, 0.6 µl of 1.2 mM MgCl_2_, 1 µl of 0.2 mM dNTPs mix, 1.25 µl of 0.5 µM of both primers, 0.1 µl of 0.5 U Taq DNA Polymerase and 1 µl of *CYP2D6* XL-PCR templates. The cycling conditions were as follows: 30 s initial denaturation at 95 °C, then 30 cycles of denaturation at 95 °C for 15 s, annealing 54 °C for 20 s, extension at 68 °C for 72 s, and a final extension step of 5 min at 68 °C. Subsequently, the presence of appropriately sized PCR products was separated on a 1.5% agarose gel electrophoresis and were purified using a FavorgenPrep™ GEL/PCR Purification Kit (Favorgen Biotech Corporation, Taiwan), followed by automated DNA sequencing at Macrogen Inc. (Korea).

### Detection of *CYP2D6* coding region mutations by direct sequencing of PCR-reamplified products (DSP)

The isolated *CYP2D6* gene, which consists of nine exons, was PCR-reamplified by nested DSP primers. The nested DSP reactions were conducted on a Mastercycler-pro (Eppendorf, Hamburg, Germany). All six different PCR mixes were done in a 25 µl reaction mixture containing 19.4 µl of nuclease-free water, 2.5 µl of 1× Master Mix buffer, 0.75 µl of 1.5 mM MgCl_2_, 1 µl of 0.2 mM dNTPs mix, 1.25 µl of 0.5 µM of each primer pair, 0.1 µl of 0.5 U Taq DNA Polymerase and 1 µl of *CYP2D6* XL-PCR template. Since reactions used similar annealing temperatures, identical cycling conditions could be used for all reactions: 30 s initial denaturation at 95 °C, then 30 cycles of denaturation at 95 °C for 15 s, annealing at 53 °C for 20 s, extension at 68 °C for 55 s, and a final extension step of 5 min at 68 °C. Identification of accurately sized PCR products, purification and sequencing were performed as mentioned earlier in the INT2 method. Sequencing was indeed performed bi-directionally, which will decrease the chance of a false-positive result. In addition, the long-range PCR tag polymerase used in the reactions has high fidelity, and a positive control was included in each batch. Finally, mutations identified are known variants, rather than random SNPs. Taken together, the reported mutations are very unlikely explained by sequencing errors.

### Rapid identification of four polymorphic loci by allele-specific oligonucleotide probes real-time SNPs genotyping (ASO)

All analyzed samples with known *CYP2D6* SNPs were genotyped in duplicate, using diluted XL-PCR fragments derived from both duplications/multiplications and deletions in order to ensure concordant SNPs calls. Genotyping for the key mutations, C100T [rs1065852], G1846A [rs3892097], C2850T [rs16947] and G4180C [rs1135840], were performed on StepOnePlus™ Real-time PCR Systems (Applied Biosystems Inc., Foster City, CA USA). The real-time PCR reactions were carried out in the final volume of 25 µl consisted of: 12.5 µl of 1× Tag Man Genotyping Master Mix (Roche Molecular Systems, Inc.), 1.25 µl of 1× mixed each forward, reverse and variant-specific probes, 10.75 µl of nuclease-free water, and 0.5 µl of XL-PCR template diluted at 3000-fold. Thermocycling conditions were as follows: 60 °C for 30 s followed by 95 °C for 10 min, followed by 40 cycles of 95 °C for 15 s, 60 °C for 1 min, and post-read stage at 60 °C for 30 s. Positive and negative controls were included in each run.

## Results

### *CYP2D6* genotyping assay

**Fig. 2:**
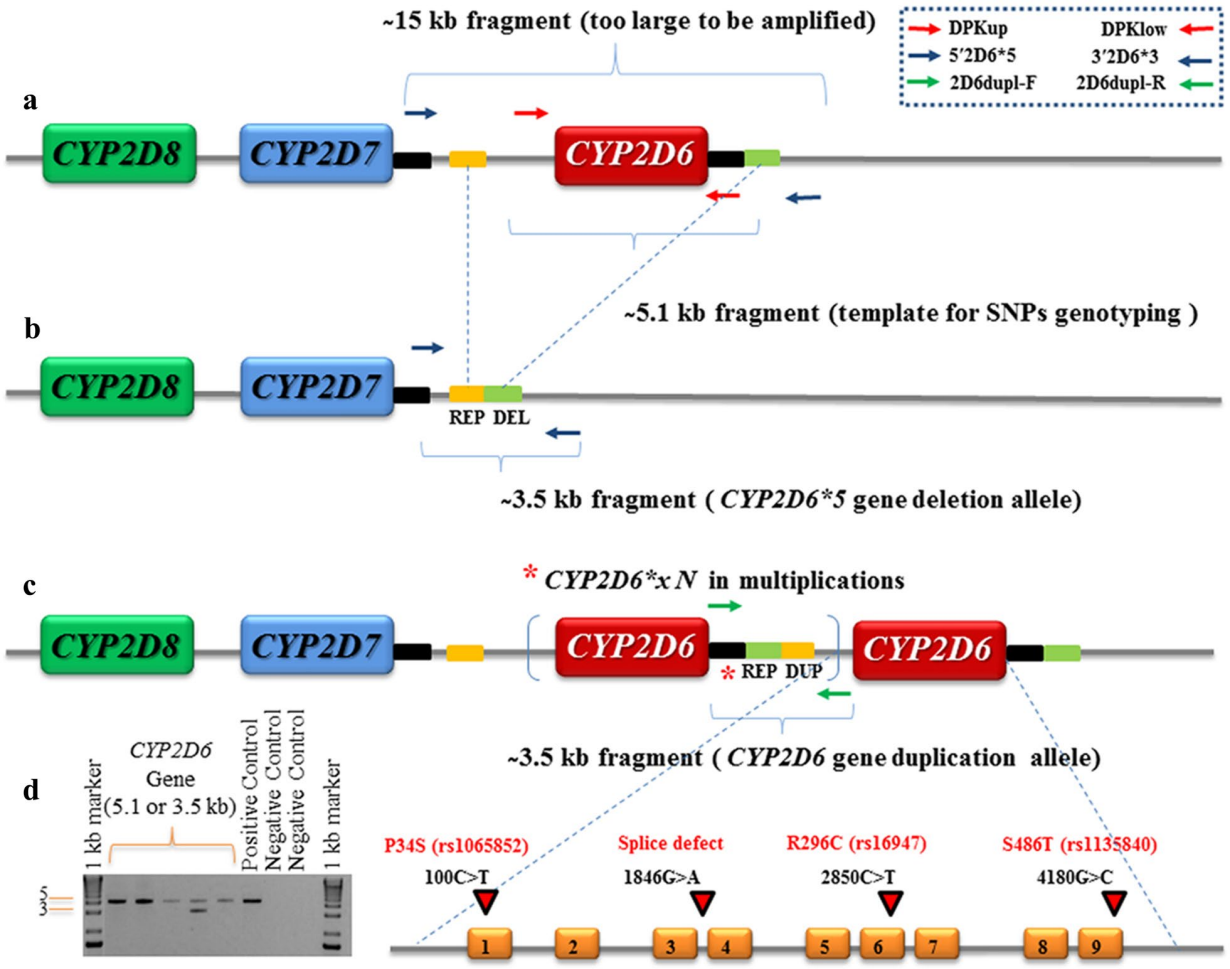
Overview of CYP2D locus arrangements and typical recombinant events. **a** A single *CYP2D6* gene is located downstream of the CYP2D locus, with two pseudogenes *CYP2D8/2D7* flanking at the 5′ end of the *CYP2D6* gene. The position of each forward and reverse pair for amplifying the target fragments are represented by different colored arrows. **b** In the CYP2D6*5 deletion allele, the entire *CYP2D6* is deleted from the CYP2D locus producing the new hybrid (REP DEL) containing 5′ yellow box sequence and 3′ green box sequence. **c** The general duplication arrangement, the hybrid REP DUP containing 5′ green box sequence and 3′ yellow box sequence were formed at 5′ of the *CYP2D6* gene in case of multiple *CYP2D6* gene copies occur **d** the 5.1 kb XL-PCR products encompass the entire *CYP2D6* gene consisting of 9 exons with the four known clinically relevant point mutations located at different polymorphic sites. The 3.5 kb long fragment represents either duplications/multiplication or deletion

Successfully produced entire *CYP2D6* fragments (XL-PCR) served as templates in the assay (Fig. 2). Full-length intron 2 sequencing (INT2), which primers anneals internally to the XL-PCR products, distinguished between *CYP2D7/8* pseudogenes and the functional *CYP2D6* gene. All nucleotide sequences of the intron 2 region showed more than 95% sequence similarity to the *CYP2D6* gene, indicating that the desired templates were generated correctly (see Additional file 1). Paired forward-reverse reads of 9 exons in each individual sample demonstrated that the sense *CYP2D6*-specific strands were perfectly matched base-by-base to their antisense strands, implying that the presence of call variants on the sequences could be identified correctly. Due to the high quality of DNA sequencing, heterozygous (double) peaks could be clearly identified, as shown in Additional file 2. Regarding the ASO genotyping assay, the seventy *CYP2D6* specific amplicons were genotyped in duplicate at four selected loci residing within the *CYP2D6* target sequence, showing good performance (see Addition file 2). The auto call analytical StepOne Software v2.1 (Applied Biosystems, Inc.) automatically generated allele discrimination plots with well-separated clusters for genotype callings, and the call rate in each assay was above 95%. All replicate assay results of individual samples were 100% identical in the variant call as they clustered in the same region of the scatterplot. Furthermore, in all seventy tested pairs of individual samples the genotyping results generated by the DSP and ASO assays showed perfect concordance for the assayed genotypes, representing the metabolic enzyme variants in the study population (see Additional file 2).

### *CYP2D6* variability in the Karen population

*CYP2D6*-specific XL-PCR products were successfully amplified in all seventy patients, of which 2 (3%) carried multiple active functional *CYP2D6* genes and 4 (6%) a whole *CYP2D6* gene deletion (Fig. 2). Combining all direct sequencing results of full-length *CYP2D6* coding regions, at least one mutation was detected in all exons except in exon 4, 5, 7 and 8. Three non-synonymous substitutions (C100T: Exon1, C2850T: Exon6 and G4180C: Exon9), two synonymous substitutions (C1039T: Exon2 and G1661C: Exon3) and a mutation at the splice junction (G1846A) were detected. Frequencies of each allele and genotype are summarized in Additional file 3. Of the six mutations identified, SNPs at loci G1661C and G4180C were most frequent (0.76), followed by SNPs at loci C100T, C1039T and C2850T (0.43, 0.43 and 0.32, respectively), whereas SNP at loci G1846A was less frequent (0.02). The homozygous mutant genotype 4180C/C was the most common, and was found in 41 individuals (0.59), followed by 100C/T in 38 (0.54), 2850C/T in 32 (0.46), 4180G/C in 22 (0.31), 100T/T in 11 (0.16) and 2850T/T in 6 (0.09). In contrast, genotype frequencies of the splice site defect 1846G/A 3 (0.04) and 1846A/A 0 (0.00) were very low. *CYP2D7* exon 9 conversion, assessed through entire exon 9 sequencing and associated with decreased enzymatic activity, was observed in only one individual (see Additional file 4). Data generated from the DSP/ASO genotyping assays, the 6 distinct alleles and 11 genotype frequencies, are summarized in Table 3. Other alleles and genotypes described in the literature were not observed in this Karen study population.

**Table 3:** Distribution of *CYP2D6* genotypes and alleles in the Karen study population

Genotypes	*n*	Genotype frequencies
*1/*1	3	0.043
*1/*2	8	0.114
*1/*4	3	0.043
*1/*5	2	0.029
*1/*10	10	0.143
*1/*36	1	0.014
*2/*2	7	0.100
*2/*5	1	0.014
*2/*10	23	0.329
*5/*10	1	0.014
*10/*10	11	0.157
Total	70	1

Alleles	*n*	Allele frequencies
*1	30	0.214
*2	46	0.329
*4	3	0.021
*5	4	0.029
*10	56	0.400
*36	1	0.007
Total	140	1

### Comparative analysis of *CYP2D6* variants in different populations

**Fig. 3:**
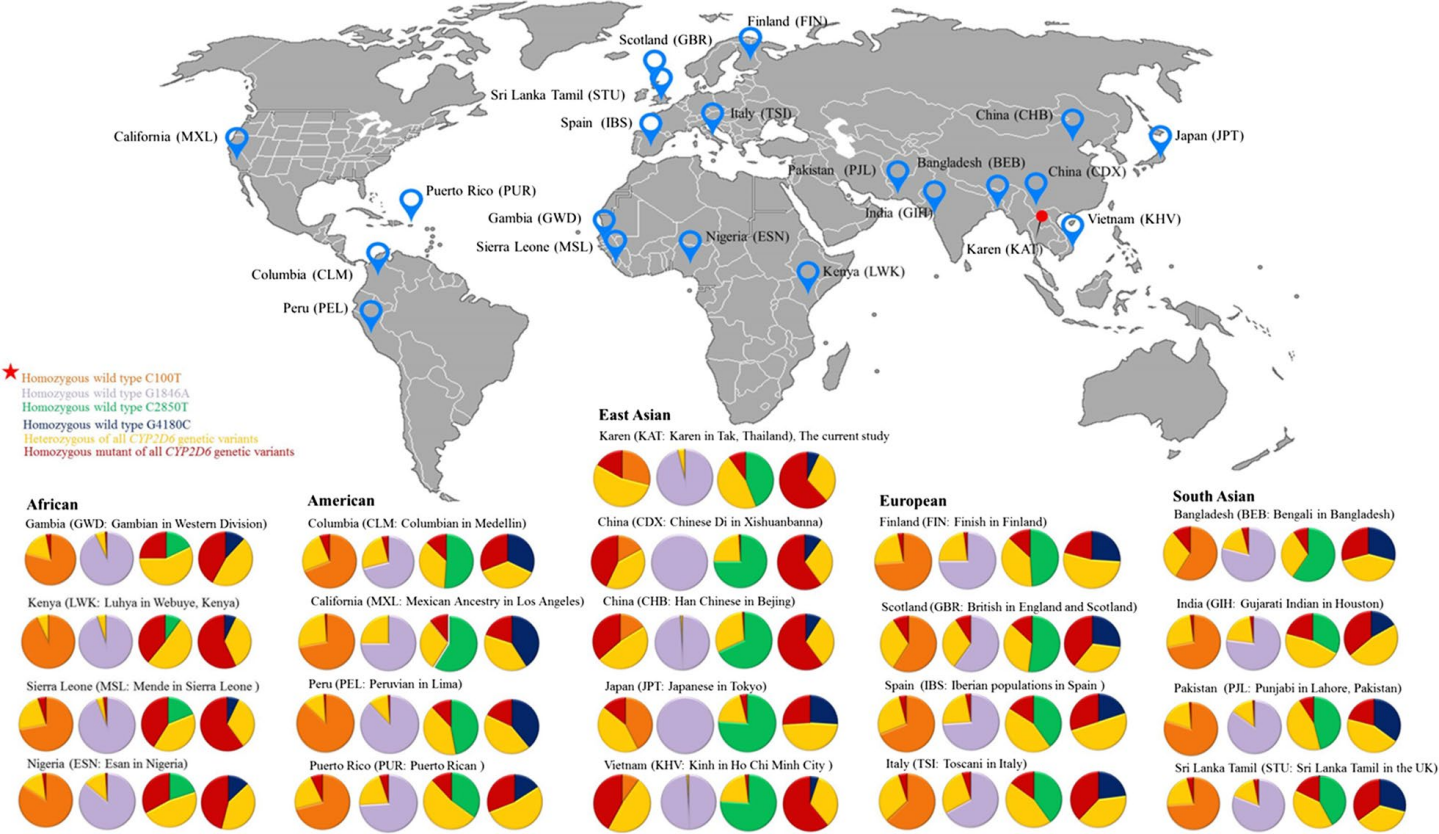
1000 Genomes project phase 3 genotype frequencies. The genotype frequencies differ considerably among the ethnic populations, as demonstrated on selected common allelic variants being present in the Karen, C100T [rs1065852], G1846A [rs3892097], C2850T [rs16947] and G4180C [rs1135840]. The obtained frequencies were from http://www.ensembl.org

The genotyping results was compared to the list of global genetic mutations in *CYP2D6* from the 1000 Genome Project (http://www.ensembl.org) (Fig. 3). Although this study was conducted in *P. vivax* infected patients, the genotypic pattern observed in the Karen study population was similar to other patterns reported from East Asia, including China, Japan and Vietnam. The exception was C2850T, which showed a notably higher frequency in this study population (Table 4). As the geographical distance increased from the study area, including Bangladesh, India, Pakistan and Sri Lanka, the similarity between genotypes decreased. Similarity in the distributions of genotype frequencies was further reduced when comparing populations in Africa, America and Europe (Fig. 3 and Additional file 5).

**Table 4:** *CYP2D6* allele frequencies in Karen compared with frequencies previously described in other ethnic populations

Population	Year	*n*	Methods	C100T P34S (*10)	C1039T	G1661C	G1846A Splicing (*4)	C2850T R296C (*2)	G4180C S486T	Gene conversion (*36)	Deletion (*5)	Duplications
Asian												
Karen (current study)	2017	70	DSP/ASO	0.40	0.43	0.76	0.02	0.33	0.76	0.01	0.03	0.01
Thai [44]	2011	286	M/DHPLC	0.45	ND	ND	0.01	0.10	ND	0.16	0.04	0.004
Thai [49]	2012	48	AmpliChip	0.44	ND	ND	0.01	0.10	ND	0.01	0.04	ND
Thai [50]	2013	114	AmpliChip	0.46	ND	ND	0.01	0.10	ND	0.01	0.04	ND
Thai [51]	2013	233	Amplichip	0.48	ND	ND	0.01	0.09	ND	0.01	0.05	0.004
Thai [45]	2016	84	Luminex xTag	0.52	ND	ND	0.01	0.06	ND	ND	ND	0.06
Chinese [52]	2002	223	ASA	0.51	ND	ND	0.002	ND	ND	ND	0.07	0.01
Chinese [53]	2008	400	Sequencing	0.53	ND	ND	0.001	0.11	ND	ND	0.05	0.02
Hong Kong Chinese [54]	2000	119	PCR–RFLP	0.65	ND	ND	0.00	0.08	ND	ND	0.05	ND
Vietnamese [55]	2010	122	mSBE	0.57	ND	ND	ND	0.08	ND	ND	0.06	0.00
Japanese [56]	1999	98	PCR	0.41	ND	ND	0.02	0.09	ND	ND	0.06	ND
Japanese [57]	2000	412	PCR–RFLP	0.38	ND	ND	0.002	0.13	ND	ND	0.05	0.01
Japanese [58]	2003	162	ASA-RFLP	0.38	ND	ND	0.00	0.13	ND	0.01	0.06	ND
Korean [59]	2006	400	Sequencing	0.45	ND	ND	0.003	0.10	ND	ND	0.06	0.01
Korean [60]	2009	758	Sequencing	0.46	ND	ND	ND	0.10	ND	ND	0.06	0.01
Korean [61]	2011	766	SNaPshot	0.43	ND	ND	0.001	0.12	ND	ND	0.06	0.01
South India [62]	2006	447	PCR–RFLP	0.10	ND	ND	0.07	0.35	ND	ND	0.02	ND
Caucasian												
Germany [63]	1997	589	PCR–RFLP	0.02	ND	ND	0.21	0.32	ND	ND	0.02	0.007
Mexican-American [64]	2001	349	PCR–RFLP	0.07	ND	ND	0.10	0.23	ND	ND	0.02	0.01
Spanish [65]	2006	105	RT-PCR	0.02	ND	ND	0.14	0.40	ND	ND	0.03	0.04
African												
African-American [66]	2001	154	PCR–RFLP	0.08	ND	ND	0.08	0.27	ND	ND	0.06	0.02
African-American [67]	2006	222	AmpliChip	0.04	ND	ND	0.07	0.05	ND	0.005	0.06	0.05
African-American [68]	2013	75	mPCR	0.03	ND	ND	0.08	0.29	ND	ND	0.09	ND

Allele frequencies of CYP2D6*2, *4, *5, *10 and *36 were calculated based on the presence of C2850T, G1846A, entire *CYP2D6* gene deletion, C100T and gene conversion, respectively

*n*, the number of subjects ND, allele not determined in reference study; DSP/ASO, direct sequencing of PCR-reamplified products and allele-specific oligonucleotide probes real-time SNPs genotyping; ASA, allele-specific amplification assay; mPCR or SBE, multiplex PCR or single-base extension; M/DHPLC, multiplex PCR coupled with semi-quantitative denaturing high-performance liquid chromatography

## Discussion

Primaquine is required for preventing *P. vivax* malaria relapses [1]. The radical curative efficacy of primaquine is thought to be mainly dependent on *CYP2D6*-mediated metabolism [12]. In addition to primaquine, its long half-life analogue tafenoquine has recently been developed for radical cure of *P. vivax* malaria. However, there are somewhat conflicting results whether differences in *CYP2D6* metabolism confers differences in therapeutic efficacy. A clinical study showed that tafenoquine efficacy in *P. vivax*-infected patients was not affected by the changes in *CYP2D6* activity [30]. In contrast, tafenoquine pharmacokinetic profiles in *CYP2D* knockout mice were differed significantly from those of wild-type mice, suggesting that tafenoquine could be possibly affected by the *CYP2D* metabolism [31]. Further studies will be needed to assess the importance of *CYP2D6* mutations in tafenoquine biological activity. Since the *CYP2D6* gene is highly polymorphic with hundreds of variant alleles described, it potentially affects a large range of clinically used drugs metabolized by the encoded enzyme [32]. Related to this, there is a growing interest for the development of user-friendly *CYP2D6* genotyping platforms with sufficiently high throughput to characterize clinically relevant genetic variations in the *CYP2D6* gene. Techniques previously described include restriction fragment length polymorphism (PCR–RFLP) [33, 34], single strand conformation polymorphism (SSCP) [35, 36], multiplex allele-specific PCR (Multiplex PCR) [37, 38], and allele-specific oligonucleotide hybridization (PCR-ASO) [39]. However, these techniques are mostly laborious to execute, time-consuming, error-prone and characterize only a limited number of alleles. Real-time PCR-based strategies enable detection of a larger number of mutations and are more rapid, but often deploy in-house developed primers with limited specificity, resulting in unwanted co-amplification of pseudogenes [40–42]. More recent techniques including pyrosequencing [43], denaturing high-performance liquid chromatography (DHPLC) [44] and Luminex-xTag [45] perform better with shorter run-times, but all require highly advanced equipment often not available in malaria endemic countries. High-throughput microarray technology, such as GeneChip CYP450, Amplichip CYP450 and the DMET microarray [46–48], have excellent performance and allele coverage, but is also technically difficult and costly.

In the current study, several steps were taken to increase performance of the assay: Firstly, the problem of co-amplification of *CYP2D* with high sequence similarity was overcome by using *CYP2D6*-specific amplification primers (XL-PCR) and a nested PCR approach (INT2). Secondly, introduction of direct sequencing of PCR-reamplified products (DSP) lowered the chance of missing non-targeted variations within the exonic *CYP2D6* sequences. Thirdly, addition of the ASO assay enabled rapid identification of four allelic sites observed in the Karen study population, requiring approximately 90 min for parallel identification. Overall, our customizable ASO assay showed high accuracy with very high SNP call rates for each genotype and absence of contamination errors. Moreover, it yields high-intensity fluorescent signals and clearly separating allelic clusters. Reproducibility of the assay was not formally assessed, since this would involve in the results generated by inter-laboratory tests. However, the DSP and ASO assays were repeatedly genotyped, at least 2 times on different days, to assure the repeatability of the assays, and were analysed simultaneously with known *CYP2D6* genotypes. The replications of *CYP2D6* genotyping results were in 100% concordance. In order to rule out contamination, negative samples, consisting of nuclease free water, were evaluated in parallel within a single analytical run together with the patients’ samples. To reduce the chance of cross-reactivity in the experiments, 3 approaches were applied (a) all generated *CYP2D6* templates were assayed by INT2 to confirm absence of cross-reactivity with the pseudogenes, (b) to minimize unintended binding to the pseudogenes, allele-specific primer and probe sets were designed using the public databases (NCBI and dbSNP), and (c) thermal cycling was optimized to reduce non-specific binding. Particularly, no significant interfering substances have been observed. Comparison of the ASO assay to the reference standard, DSP sequencing, showed full concordance between the two methods. Therefore, the customizable ASO assay is a promising tool for large-scale studies because it is simple, requiring limited processing, reducing the risk for contamination and showing good performance at reasonable costs. Sequencing the purified PCR products with 500–1000 bp coverage of the target site by Macrogen Inc., Korea, costs approximately 3.5 GBP/point mutation. 1 ml of Tag Man Genotyping Master mix, which is approximately 70 GBP, can be used for 70 reactions (12.5 μl/reaction), which translates to around 1 GBP/point mutation. The rough cost comparison of using ASO against a gold standard test revealed that all ASO reagents identified a single point mutation are relatively inexpensive and cost three times cheaper. Compared to the previous published TagMan based *CYP2D6* genotyping assays, the here described assay has better performance by lacking cross-reactivity with the two pseudogenes *CYP2D7* and *CYP2D8*. A limitation of the assay is the interpretation of results in heterozygous individuals with gene multiplications; the current method does not assess specific gene allele duplication or quantify the copy number for each allele. However, whereas gene amplification might result in increased production of the active metabolite of primaquine potentially increasing its efficacy, reduced activity of the drug is mainly associated with gene mutations resulting in reduced *CYP2D6* activity. The latter was the scope of the study.

Initial use of the assay showed that the *CYP2D6* allelic frequencies in this vivax-infected population corresponded well with the observed frequencies in nearby Asian populations, but were different from frequencies in Caucasian and African populations. This similarity suggests absence of selective pressure on the *CYP2D6* genotype in this *P. vivax* infected population. The most abundant allele in the Karen population was the CYP2D6*10 allele, occurred at a frequency of 0.40, suggested that there is a high prevalence of individuals with reduced metabolic capacity for *CYP2D6* dependent substrates. Whereas the CYP2D6*4 and *5 defective alleles, occurred at a frequency of 0.02 and 0.03 respectively, represents rare causes of reduced enzyme activity in this population. The CYP2D6*2 allele with a frequency of 0.33 was the most common functional allele in the Karen population, with high frequency of the 2850C/T and 2850T/T alleles. The number of *CYP2D7* exon 9 conversion carriers (CYP2D6*36) was very small in the study, and a larger study is warranted to assess the frequency of this clinically important genotype in more detail. Since *CYP2D6* allele frequencies vary markedly across ethnic populations, the ASO assay would have to be re-evaluated for other geographical areas. Additional file 1 shows the differences in *CYP2D6* allele frequencies present in other areas including Africa, America, Europe and South Asian. The DSP approach providing the sequence the *CYP2D6* region of the population of interest could inform which adaptations in a customized ASO assay would be necessary. Further studies are planned on *CYP2D6* mutations in the Karen population using the ASO genotyping platform. Results will be compared to the data on the efficacy of primaquine in the study cohort to inform if efficacy proves to be compromised by *CYP2D6* mutations associated with decreased *CYP2D6* enzyme activity. Indeed, this is not a point of care test, but it is a simple method, which is easy to set up in molecular laboratories in tropical countries. Defining the impact of *CYP2D6* mutations on primaquine dosing and efficacy will require a clinical trial, which can make use of the platform described here. The prevalence of mutation associated with defective *CYP2D6* phenotypes in the Karen population has prompted our group to initiate such a study. Elimination of *P. vivax* will require wider deployment of radical cure with primaquine in an effective dose. This effective dose might differ according to the prevalent *CYP2D6* mutations in the population. This relationship should be studied more extensively in different populations to ensure proper dosing. The described technique could facilitate this.

## Conclusion

The ASO assay is a new *CYP2D6* genotyping assay with high-accuracy and high-reproducibility for the detection of common *CYP2D6* variant alleles, and is suitable for large-scale surveys. The high prevalence in the *P. vivax* infected patients in Karen population of the CYP2D6*10 allelic variant associated with reduced *CYP2D6* enzyme activity could potentially affect the radical curative efficacy of primaquine and warrants more extensive evaluation.

## Additional files

**Additional file 1: Figure S1.** Discrimination of functional and non-functional genes using intron 2 sequencing. The multiple sequence alignment of Intron 2 region (1.1 kb) of each CYP2D gene compared among individual’s intron 2 sequence.

**Additional file 2: Figure S2.** Comparison of the two genotyping platforms. DSP (electropherograms) and ASO (amplification plot) detected the presence of four common genetic variations in the *CYP2D6* gene including C100T, G1846A, C2850T and G4180C. The X axis of the amplification plot shows the relative fluorescence for wild-type alleles (green curve) and variant alleles (blue curve), respectively. Clusters of the homozygous wild-type, heterozygous and homozygous mutant are also shown in the allelic discrimination plots (NTC = no template control). The ASO genotyping results were in accordance with the results obtained by DSP.

**Additional file 3: Figure S3.** The allele and genotype frequencies from different *CYP2D6* coding regions. The bottom panel illustrates the successful amplification of exonic *CYP2D6*-specific PCR fragments uniquely generated by the DSP assay. Five colored bars are shown for each exon, representing the genotype frequency of homozygous wild-type (orange), heterozygous (gray), homozygous mutant (blue), heterozygous deletion (X, green) wild-type, and heterozygous deletion (X, red) mutant. The allele frequencies are presented on top; deletion alleles were carried in each variant with a frequency of 0.03 (*D (X), red). Analysis used STATA/SE12.1 to calculate frequencies.

**Additional file 4: Figure S4.** Evidence of gene conversion to *CYP2D7* in the exon 9 of the *CYP2D6* gene. Red boxes denote the converted region, the 5′ end of the *CYP2D6* gene fused with the 3′ end of *CYP2D7* gene as a result of partial gene recombination, located at the downstream position of the exon 9.

**Additional file 5: Table S1.** 1000 Genomes project phase 3 genotype frequencies.
