# Supplementary material for: Real time PCR detection of common CYP2D6 genetic variants and its application in a Karen population study
Source: Malar J. 2018 Nov 15;17:427. doi: 10.1186/s12936-018-2579-8 (PMC6238304; doi:10.1186/s12936-018-2579-8)
Supplement: Supplementary file 1 — Additional file 1: Figure S1. Discrimination of functional and non-functional genes using intron 2 sequencing. The multiple sequence alignment of Intron 2 region (1.1 kb) of each CYP2D gene compared among individual’s intron 2 sequence. [file 12936_2018_2579_MOESM1_ESM.docx]

**
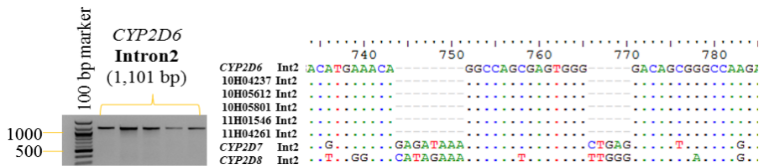
**

Additional file 1: Figure S1. Discrimination of functional and non-functional genes using intron 2 sequencing. The multiple sequence alignment of Intron 2 region (1.1kb) of each CYP2D gene compared among individual’s intron 2 sequence.
