# Supplementary material for: Real time PCR detection of common CYP2D6 genetic variants and its application in a Karen population study
Source: Malar J. 2018 Nov 15;17:427. doi: 10.1186/s12936-018-2579-8 (PMC6238304; doi:10.1186/s12936-018-2579-8)
Supplement: Supplementary file 2 — Additional file 2: Figure S2. Comparison of the two genotyping platforms. DSP (electropherograms) and ASO (amplification plot) detected the presence of four common genetic variations in the CYP2D6 gene including C100T, G1846A, C2850T and G4180C. The X axis of the amplification plot shows the relative fluorescence for wild-type alleles (green curve) and variant alleles (blue curve), respectively. Clusters of the homozygous wild-type, heterozygous and homozygous mutant are also shown in the allelic discrimination plots (NTC = no template control). The ASO genotyping results were in accordance with the results obtained by DSP. [file 12936_2018_2579_MOESM2_ESM.docx]

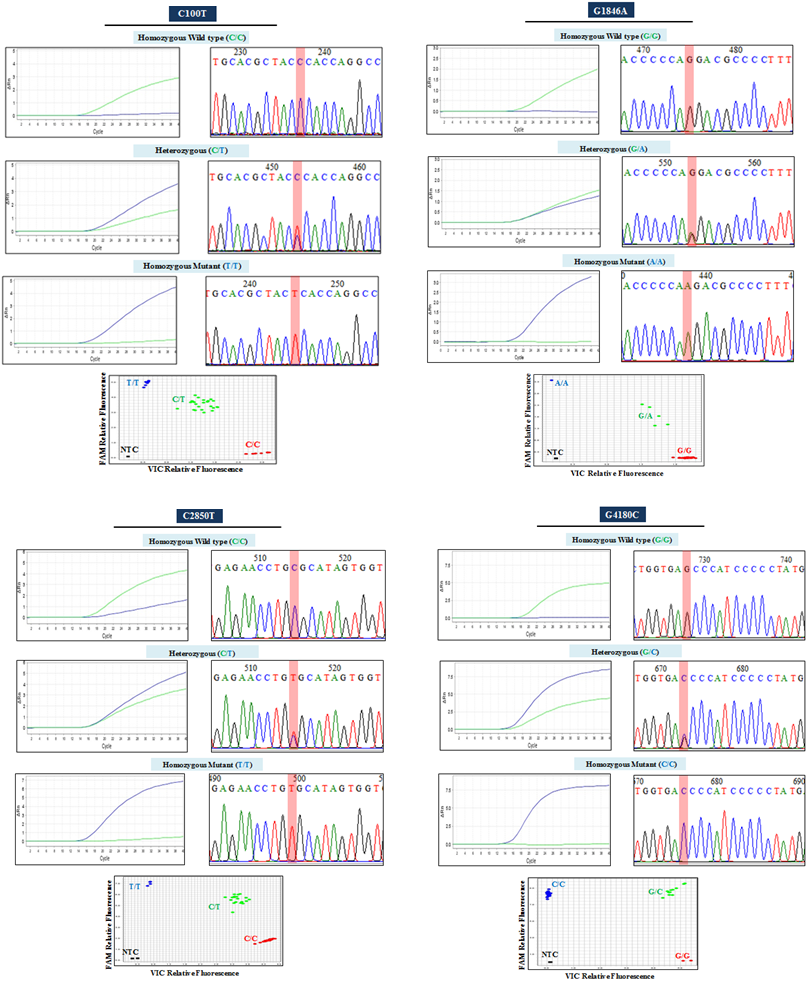


Additional file 2: Figure S2. Comparison of the two genotyping platforms. DSP (electropherograms) and ASO (amplification plot) detected the presence of four common genetic variations in the *CYP2D6* gene including C100T, G1846A, C2850T and G4180C. The X axis of the amplification plot shows the relative fluorescence for wild-type alleles (green curve) and variant alleles (blue curve), respectively. Clusters of the homozygous wild-type, heterozygous and homozygous mutant are also shown in the allelic discrimination plots (NTC=no template control). The ASO genotyping results were in accordance with the results obtained by DSP.
