# Supplementary material for: Real time PCR detection of common CYP2D6 genetic variants and its application in a Karen population study
Source: Malar J. 2018 Nov 15;17:427. doi: 10.1186/s12936-018-2579-8 (PMC6238304; doi:10.1186/s12936-018-2579-8)
Supplement: Supplementary file 3 — Additional file 3: Figure S3. The allele and genotype frequencies from different CYP2D6 coding regions. The bottom panel illustrates the successful amplification of exonic CYP2D6-specific PCR fragments uniquely generated by the DSP assay. Five colored bars are shown for each exon, representing the genotype frequency of homozygous wild-type (orange), heterozygous (gray), homozygous mutant (blue), heterozygous deletion (X, green) wild-type, and heterozygous deletion (X, red) mutant. The allele frequencies are presented on top; deletion alleles were carried in each variant with a frequency of 0.03 (*D (X), red). Analysis used STATA/SE12.1 to calculate frequencies. [file 12936_2018_2579_MOESM3_ESM.docx]

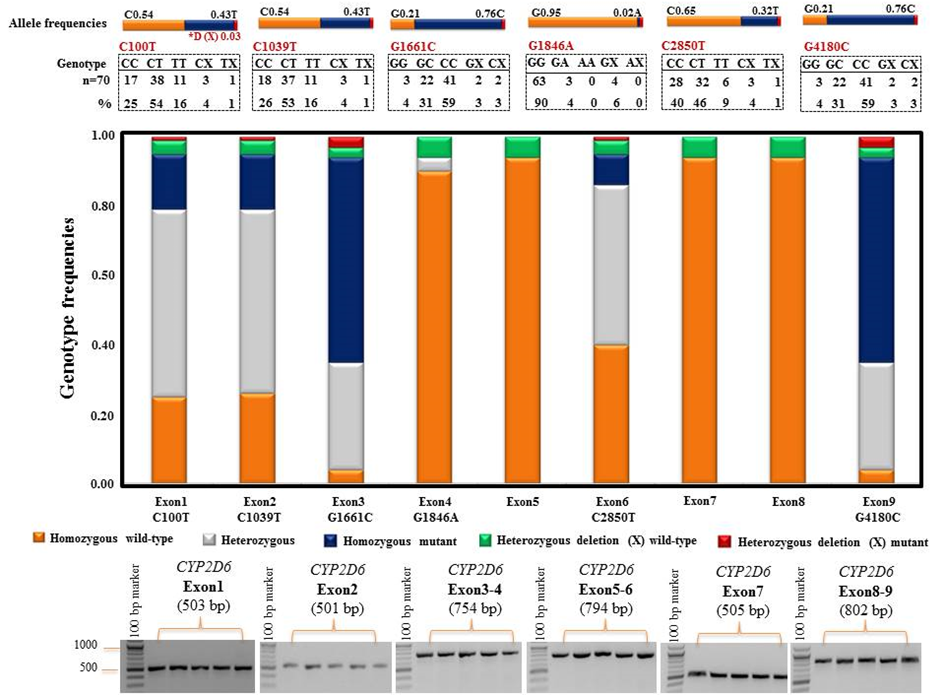


Additional file 3: Figure S3. The allele and genotype frequencies from different *CYP2D6* coding regions. The bottom panel illustrates the successful amplification of exonic *CYP2D6*-specific PCR fragments uniquely generated by the DSP assay. Five colored bars are shown for each exon, representing the genotype frequency of homozygous wild-type (orange), heterozygous (gray), homozygous mutant (blue), heterozygous deletion (X, green) wild-type, and heterozygous deletion (X, red) mutant. The allele frequencies are presented on top; deletion alleles were carried in each variant with a frequency of 0.03 (*D (X), red). Analysis used STATA/SE12.1 to calculate frequencies.
