# Supplementary material for: Real time PCR detection of common CYP2D6 genetic variants and its application in a Karen population study
Source: Malar J. 2018 Nov 15;17:427. doi: 10.1186/s12936-018-2579-8 (PMC6238304; doi:10.1186/s12936-018-2579-8)
Supplement: Supplementary file 4 — Additional file 4: Figure S4. Evidence of gene conversion to CYP2D7 in the exon 9 of the CYP2D6 gene. Red boxes denote the converted region, the 5′ end of the CYP2D6 gene fused with the 3′ end of CYP2D7 gene as a result of partial gene recombination, located at the downstream position of the exon 9. [file 12936_2018_2579_MOESM4_ESM.docx]

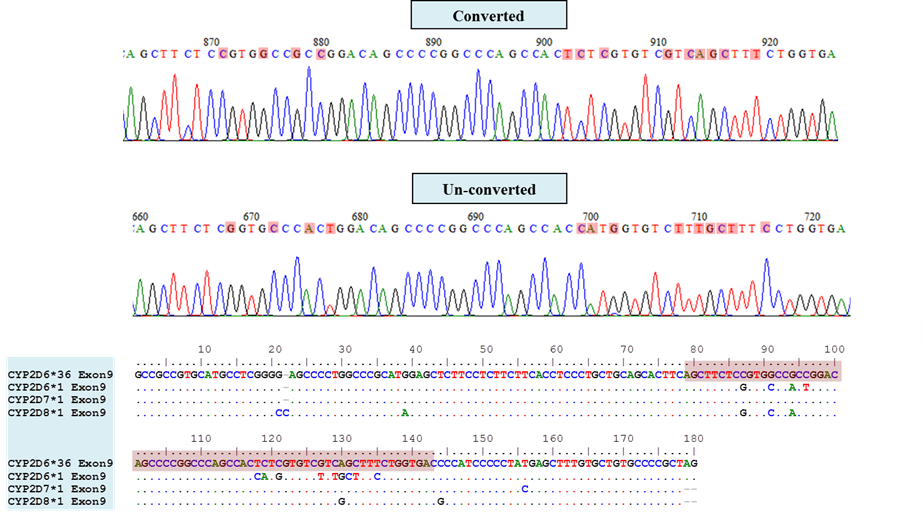


Additional file 4: Figure S4. Evidence of gene conversion to *CYP2D7* in the exon 9 of the *CYP2D6* gene. Red boxes denote the converted region, the 5′ end of the *CYP2D6* gene fused with the 3′ end of *CYP2D7* gene as a result of partial gene recombination, located at the downstream position of the exon 9.
