# Supplementary material for: Real time PCR detection of common CYP2D6 genetic variants and its application in a Karen population study
Source: Malar J. 2018 Nov 15;17:427. doi: 10.1186/s12936-018-2579-8 (PMC6238304; doi:10.1186/s12936-018-2579-8)
Supplement: Supplementary file 5 — Additional file 5: Table S1. 1000 Genomes project phase 3 genotype frequencies. [file 12936_2018_2579_MOESM5_ESM.docx]

**Additional file 5: Table S1. 1000 Genomes project phase 3 genotype frequencies**

| **Genotype Frequencies** | **C100T**  **(** rs1065852**)** | | | **G1846A**  **(** rs3892097**)** | | | **C2850T**  **(** rs16947**)** | | | **G4180C**  **(** rs1135840**)** | | |
| --- | --- | --- | --- | --- | --- | --- | --- | --- | --- | --- | --- | --- |
|  | **C/C** | **C/T** | **T/T** | **G/G** | **G/A** | **A/A** | **C/C** | **C/T** | **T/T** | **G/G** | **G/C** | **C/C** |
| ***Karen (*n*=70)** | **0.25** | **0.54** | **0.16** | **0.90** | **0.04** | **0.00** | **0.40** | **0.46** | **0.09** | **0.04** | **0.31** | **0.59** |
| **All** | **0.63** | **0.27** | **0.10** | **0.84** | **0.14** | **0.02** | **0.45** | **0.39** | **0.16** | **0.20** | **0.41** | **0.39** |
| **African** | **0.80** | **0.17** | **0.03** | **0.89** | **0.10** | **0.01** | **0.22** | **0.46** | **0.32** | **0.13** | **0.39** | **0.48** |
| **ACB** | **0.74** | **0.23** | **0.03** | **0.84** | **0.14** | **0.02** | **0.32** | **0.37** | **0.31** | **0.18** | **0.36** | **0.46** |
| **ASW** | **0.74** | **0.21** | **0.05** | **0.79** | **0.18** | **0.03** | **0.33** | **0.51** | **0.16** | **0.20** | **0.46** | **0.34** |
| **ESN** | **0.84** | **0.14** | **0.02** | **0.86** | **0.13** | **0.01** | **0.20** | **0.47** | **0.33** | **0.13** | **0.41** | **0.46** |
| **GWD** | **0.79** | **0.18** | **0.03** | **0.93** | **0.06** | **0.01** | **0.18** | **0.57** | **0.25** | **0.12** | **0.46** | **0.42** |
| **LWK** | **0.93** | **0.07** | **0.00** | **0.95** | **0.05** | **0.00** | **0.10** | **0.51** | **0.39** | **0.07** | **0.36** | **0.57** |
| **MSL** | **0.72** | **0.23** | **0.05** | **0.94** | **0.04** | **0.02** | **0.19** | **0.40** | **0.41** | **0.07** | **0.33** | **0.60** |
| **YRI** | **0.81** | **0.17** | **0.02** | **0.89** | **0.11** | **0.00** | **0.24** | **0.40** | **0.36** | **0.15** | **0.35** | **0.50** |
| **American** | **0.75** | **0.21** | **0.04** | **0.77** | **0.20** | **0.03** | **0.47** | **0.41** | **0.12** | **0.30** | **0.44** | **0.26** |
| **CLM** | **0.69** | **0.25** | **0.06** | **0.71** | **0.25** | **0.04** | **0.51** | **0.36** | **0.13** | **0.32** | **0.37** | **0.31** |
| **MXL** | **0.72** | **0.27** | **0.01** | **0.75** | **0.25** | **0.00** | **0.59** | **0.30** | **0.11** | **0.41** | **0.39** | **0.20** |
| **PEL** | **0.87** | **0.12** | **0.01** | **0.88** | **0.11** | **0.01** | **0.47** | **0.41** | **0.12** | **0.39** | **0.43** | **0.18** |
| **PUR** | **0.71** | **0.22** | **0.07** | **0.74** | **0.21** | **0.05** | **0.35** | **0.53** | **0.12** | **0.16** | **0.53** | **0.31** |
| **East Asian** | **0.21** | **0.43** | **0.36** | **0.99** | **0.01** | **0.00** | **0.74** | **0.23** | **0.03** | **0.12** | **0.36** | **0.52** |
| **CDX** | **0.17** | **0.40** | **0.43** | **1.00** | **0.00** | **0.00** | **0.75** | **0.24** | **0.01** | **0.10** | **0.30** | **0.60** |
| **CHB** | **0.16** | **0.47** | **0.36** | **0.99** | **0.01** | **0.00** | **0.68** | **0.31** | **0.01** | **0.09** | **0.31** | **0.60** |
| **CHS** | **0.19** | **0.39** | **0.42** | **1.00** | **0.00** | **0.00** | **0.77** | **0.19** | **0.04** | **0.09** | **0.34** | **0.57** |
| **JPT** | **0.42** | **0.43** | **0.14** | **1.00** | **0.00** | **0.00** | **0.76** | **0.20** | **0.04** | **0.26** | **0.48** | **0.26** |
| **KHV** | **0.10** | **0.48** | **0.42** | **0.99** | **0.01** | **0.00** | **0.76** | **0.22** | **0.02** | **0.05** | **0.34** | **0.61** |
| **European** | **0.65** | **0.30** | **0.05** | **0.67** | **0.28** | **0.05** | **0.46** | **0.39** | **0.15** | **0.23** | **0.44** | **0.33** |
| **CEU** | **0.57** | **0.38** | **0.05** | **0.60** | **0.35** | **0.05** | **0.52** | **0.32** | **0.16** | **0.22** | **0.43** | **0.35** |
| **FIN** | **0.74** | **0.23** | **0.03** | **0.75** | **0.23** | **0.02** | **0.49** | **0.38** | **0.13** | **0.26** | **0.53** | **0.21** |
| **GBR** | **0.59** | **0.32** | **0.09** | **0.60** | **0.31** | **0.09** | **0.52** | **0.35** | **0.13** | **0.27** | **0.34** | **0.39** |
| **IBS** | **0.69** | **0.27** | **0.04** | **0.74** | **0.23** | **0.03** | **0.40** | **0.44** | **0.16** | **0.20** | **0.50** | **0.30** |
| **TSI** | **0.63** | **0.32** | **0.05** | **0.67** | **0.28** | **0.05** | **0.40** | **0.45** | **0.15** | **0.23** | **0.39** | **0.38** |
| **South Asian** | **0.71** | **0.25** | **0.04** | **0.80** | **0.17** | **0.03** | **0.43** | **0.42** | **0.15** | **0.26** | **0.43** | **0.31** |
| **BEB** | **0.59** | **0.30** | **0.11** | **0.79** | **0.17** | **0.04** | **0.59** | **0.31** | **0.09** | **0.29** | **0.42** | **0.29** |
| **GIH** | **0.72** | **0.26** | **0.02** | **0.77** | **0.21** | **0.02** | **0.33** | **0.46** | **0.21** | **0.17** | **0.47** | **0.36** |
| **ITU** | **0.69** | **0.28** | **0.03** | **0.80** | **0.17** | **0.03** | **0.36** | **0.47** | **0.17** | **0.19** | **0.48** | **0.33** |
| **PJL** | **0.80** | **0.19** | **0.01** | **0.85** | **0.14** | **0.01** | **0.46** | **0.45** | **0.09** | **0.35** | **0.44** | **0.21** |
| **STU** | **0.74** | **0.22** | **0.04** | **0.81** | **0.16** | **0.03** | **0.42** | **0.40** | **0.18** | **0.29** | **0.36** | **0.35** |

**African:** ACB=African Caribbean in Barbados, ASW=African Ancestry in Southwest US, ESN=Esan in Nigeria, GWD=Gambian in Western Division, the Gambian, LWK=Luhya in Webuye, Kenya, MSL=Mende in Sierra Leone, YRI=Yoruba in Ibadan, Nigeria

**American**: CLM=Columbian in Medellin, Columbia, MXL=Mexican Ancestry in Los Angeles, California, PEL=Peruvian in Lima, Peru, PUR=Puerto Rican in Puerto Rico

**East Asian:** CDX=Chinese Di in Xishuanbanna, China CHB=Han Chinese in Bejing, China, CHS=Southern Han Chinese, China, JPT=Japanese in Tokyo, Japan, KHV=Kinh in Ho Chi Minh City, Vietnam

**European:** CEU=Utah residents with Northern and Western European ancestry, FIN=Finish in Finland, GBR=British in England and Scotland, IBS=Iberian populations in Spain, TSI=Toscani in Italy

**South Asian**: BEB=Bengali in Bangladesh, GIH=Gujarati Indian in Houston, TX, ITU=Indian Telugu in the UK, PJL=Punjabi in Lahore, Pakistan, STU=Sri Lanka Tamil in the UK

*The genotype frequencies of the current population were calculated by including the *CYP2D6*5*, with a genotype frequency of 0.06. The obtained frequencies were from [www.ensembl.org](http://www.ensembl.org).
